# Dynamic turbulence mitigation for long-range imaging in the presence of large moving objects

**DOI:** 10.1186/s13640-018-0380-9

**Published:** 2019-01-03

**Authors:** Robert Nieuwenhuizen, Judith Dijk, Klamer Schutte

**Affiliations:** 0000 0001 0208 7216 grid.4858.1 TNO, PO Box 96864, 2509 JG The Hague, The Netherlands

**Keywords:** Turbulence, Software, Algorithms, Motion estimation, Optical flow, Video stabilization

## Abstract

Long-range imaging with visible or infrared observation systems is typically hampered by atmospheric turbulence. Software-based turbulence mitigation methods aim to stabilize and sharpen such recorded image sequences based on the image data only. Although successful restoration has been achieved on static scenes in the past, a significant challenge remains in accounting for moving objects such that they remain visible as moving objects in the output. Here, we investigate a new approach for turbulence mitigation on background as well as large moving objects under moderate turbulence conditions. In our method, we apply and compare different optical flow algorithms to locally estimate both the apparent and true object motion in image sequences and subsequently apply dynamic super-resolution, image sharpening, and newly developed local stabilization methods to the aligned images. We assess the use of these stabilization methods as well as a new method for occlusion compensation for these conditions. The proposed methods are qualitatively evaluated on several visible light recordings of real-world scenes. We demonstrate that our methods achieve a similar image quality on background elements as our prior methods for static scenes, but at the same time obtain a substantial improvement in image quality and reduction in image artifacts on moving objects. In addition, we show that our stabilization and occlusion compensation methods can be robustly used for turbulence mitigation in imagery featuring complex backgrounds and occlusion effects, without compromising the performance in less challenging conditions.

## Introduction

Atmospheric turbulence often hampers long-range imaging with visible or infrared observation systems. This turbulence causes random spatiotemporal variations in the density and consequently the local refractive index of the air between the observer and the imaged scene [1, 2]. Consequently, the recorded imagery suffers from random shifts and blurs that vary across the field of view and over time, which complicate their utility for visual detection, recognition, and identification at large distances. Software-based turbulence mitigation methods aim to restore such recorded image sequences based on the image data only [3–14]. The goal of these algorithms is to produce sharp and stable imagery of the observed scene, thus enabling visual detection, recognition, and identification at larger distances.

Although successful restoration has been achieved on static scenes in the past [3–7], there remains a significant challenge in restoring imagery depicting moving objects. The main challenge here is how to distinguish between true motion of moving objects and apparent motion due to turbulence [8–14]. Under medium turbulence conditions, the apparent shifts will have similar magnitudes and length scales as the observed frame-to-frame object motion. Medium turbulence conditions are roughly defined here as having 0.2 < *r*_0_/*D* < 1, with *D* the diameter of the observation system’s aperture and *r*_0_ the Fried parameter for the image formation:

1 $$ {r}_0={\left[0.423\ {\left(\frac{2\uppi}{\uplambda}\right)}^2{\int}_{z=0}^{z=L}{C}_n^2(z){\left(\frac{z}{L}\right)}^{5/3} dz\right]}^{-3/5}, $$

where *λ* is the wavelength, ${C}_n^2$ is the refractive index structure parameter, and *z* is the distance from the imaged object at *z =* 0 to the camera at *z = L.* Under these conditions, the apparent motion of image points due to turbulence is typically significant compared to the pixel size and compared to the size of the point spread function (PSF), but the PSF is still dominated by a central lobe, so the turbulence-degraded image still appears like a continuous deformation of the unperturbed scene followed by some blur.

Several methods have been published in the literature that deal with the challenge of detecting moving objects in turbulence-degraded image sequences. Fishbain et al. proposed a method involving temporal median filtering per pixel to obtain a reference frame [8]. Pixels in subsequent frames are then classified as belonging to background or a moving object based on the magnitude and directional variability of the estimated local displacements relative to the reference frame. Output frames are computed by taking a weighted average of the reference frame and the raw frame in each pixel, with weights corresponding to the confidence that a pixel belongs to the background.

Similarly, Chen et al. compute a reference frame as a moving average of previous frames and threshold the estimated local displacements relative to the reference frame to identify tentative moving objects [9]. Object tracking is subsequently used to filter the tentative moving objects based on track properties. Moving object pixels in the reference frame are then replaced by the raw pixel values to obtain an output frame.

Halder et al. proposed a method in which a geometrically stabilized background estimate is obtained by estimating the motion trajectories of pixels in a reference frame and taking the centroid of the trajectory as a reference position. Frames are then warped to this geometric reference and averaged. Moving objects are identified based on their intensity difference with respect to this reference and replaced by their raw pixel values [11].

Oreifej et al. proposed a very different approach, in which a three-term low-rank matrix decomposition approach is applied to decompose a sequence into three components: background, moving objects, and residual errors due to apparent motions caused by turbulence [12]. Although the method combines sensitive moving object detection with accurate turbulence mitigation on the background, it is computationally expensive and requires sparsity in the number of pixels containing moving objects. This may be prohibitive for application to sequences containing large moving objects.

Recently, Anantrasirichai et al. proposed an approach which combines moving object detection and tracking with a recursive image fusion scheme which uses the dual tree complex wavelet transform (DT-CWT) [13]. This DT-CWT fusion scheme has been shown to produce sharper fusion results than the typical multi-frame averaging employed by competing methods. Interestingly, this fusion is employed on both background and on moving objects. However, a downside of this approach is that it still makes an explicit distinction between a static background and moving objects in the registration and fusion steps.

Finally, Li proposed to compute motion fields between successive frames to warp pixels from previous frames to the current frame and average them to accomplish noise reduction [14]. Pixels in the averaged frame are then warped to the centroid position of their estimated motion trajectory in a fixed number of preceding frames. This serves as a similar geometric reference as used by Halder et al. However, moving objects are not explicitly detected. Instead, moving object pixels are similarly compensated for turbulence-induced degradation as background pixels. However, the temporal averaging employed for this compensation results in blurring of both background and moving object pixels. Also, the centroid position of pixel trajectories may cause rapidly moving objects to exhibit a substantial lag in their position in the output images relative to the raw input images.

In general, inaccurate detection and segmentation of these moving objects may lead to several problems. On the one hand, moving objects classified as background may result in motion blur on these objects. Also, larger moving objects may affect the estimation of global background motion. On the other hand, if image patches containing moving objects are not corrected for turbulence in the same manner as the background, then incorrectly classified background pixels will retain their turbulence-induced distortions. Moreover, it should be noted that in the use of many long-range camera systems, the operationally most interesting elements in the scene are most often the moving objects, which favors methods providing optimized performance on moving objects.

In this paper, we propose how turbulence mitigation can be achieved in image sequences on both the background and on large moving objects, i.e., moving objects that have linear dimensions on the order of tens of pixels or more such that their motion can be accurately determined using optical flow. Similar to the approach taken by Li, apparent motion from turbulence is compensated for in both the background and moving object pixels. However, similar to our global turbulence mitigation method for low turbulence [4], additional processing steps are used in our newly proposed method for noise reduction and compensation for blurring effects. Moreover, we propose novel methods for stabilization to prevent lags in moving object positions and we propose a means to account for occlusion effects, unlike previous methods. The considered motion estimation and stabilization methods do not employ a fixed reference frame. Therefore, the proposed turbulence mitigation methods are compatible with applications where a moving camera is used. We evaluate our approach using visible light recordings of a variety of real-word scenes.

The main contributions of this paper can be summarized as follows:

1. A new method for software-based turbulence mitigation on both background and large moving objects in medium turbulence conditions, which substantially improves upon our previous global turbulence mitigation method.
2. A comparison of three optical flow algorithms on their performance for turbulence mitigation.
3. Two new algorithms for stabilization in the presence of dynamic scene deformations due to turbulence in image sequences.
4. A new method to compensate for artifacts which may arise in our processing chain due to occlusions in the raw image sequences.

This paper is organized as follows. In Section 2, we outline the proposed method. Subsequently, Section 3 shows the turbulence mitigation results we obtained by qualitatively comparing imagery before and after our turbulence mitigation. Section 4 discusses the stability of the turbulence mitigated videos. In Section 5, we provide a comparison of the performance of the different choices for motion estimation, motion stabilization, and occlusion compensation on the different datasets in Sections 3 and 4 and we show results of a small-scale observer trial to independently qualify this performance. In Section 6, we compare the results of the proposed method to those obtained with the state-of-the-art approaches of Fishbain [8] and Oreifej [12]. Finally, Section 7 provides a general conclusion.

## Methods

**Fig. 1:**
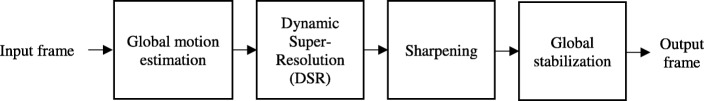
Flow diagram of the TNO global turbulence mitigation method for low turbulence (i.e., r_0_ ≿ D)

The starting point for our method is our global turbulence mitigation method for low turbulence [4]. A flowchart of this method is depicted in Fig. 1.

In this method, the first step is the estimation of the global motion (i.e., translation and rotation) of the image background between subsequent frames, where a gradient-based shift estimator [15] is used which takes the full frames as inputs. This global motion can be caused by both the average turbulence-induced motion and by the physical movements of the image sensor.

Subsequently, dynamic super-resolution (DSR) [16] is applied to the aligned images. DSR is a proprietary algorithm which estimates the true scene image that minimizes the difference between the camera images and the warped and backprojected true scene image. The backprojection employs a PSF model consisting of a Gaussian with a width parameter σ = 0.3 low-resolution pixels and rectangular camera response with 81% area fill factor per pixel. The DSR step effectively temporally averages small-scale turbulence-induced shifts of the scene over ten frames, which results in blurring of the output of the DSR step compared to the input. In the DSR step, moving objects are detected using the per-frame differences between camera images and backprojected true scene image mentioned above. All pixels are classified as either background or moving object pixels. Pixels corresponding to moving objects are not temporally averaged, but instead the pixel values from the input frame are used for the DSR output. For a given frame, the image points in the output of the DSR step are aligned with the corresponding points in the raw input frame. For example, a window in the output of the DSR step is located in the same location as in the same window in the corresponding input image, albeit on a higher resolution pixel grid.

The subsequent sharpening step reduces the turbulence-induced blur in the DSR output. Specifically, a Laplacian of Gaussian filter with σ = 1 is applied to the DSR output image, after which the filter output is multiplied by a constant and added to the DSR output image. Finally, the output is stabilized to compensate for the turbulence generated global motion by moving average filtering of the frame-to-frame motion.

Under low turbulence conditions, moving objects can be distinguished from the background by monitoring the difference between the current input frame and a temporally averaged background estimate. However, under stronger turbulence conditions, this approach breaks down because the local shifts due to turbulence lead to differences between the input frame and the background estimates all over the field of view. Previously, we have shown for sea scenarios that this can sometimes be overcome when the interesting part of the scene consists of a single moving foreground object, for example a ship [17]. In that case, the apparent motion of the object can be determined instead of its relatively uninteresting sea background, and there is no need to distinguish the object from the background to correct for the turbulence. However, this is only possible when the sea exhibits little image structure that affects the motion estimation, such as wake or waves. Moreover, in our previous approach, only simple models for the apparent motion of ships were applied, such as a global translation-rotation model. These models fail when ships exhibit more complex rotational movement due to waves.

**Fig. 2:**
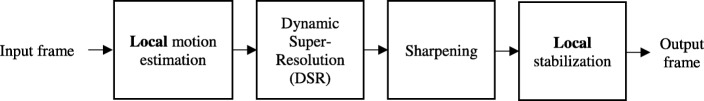
Flow diagram of the proposed method for dynamic turbulence mitigation under medium turbulence conditions (0.2 < *r*_0_/D < 1)

Here, we propose a general approach to accomplish dynamic turbulence mitigation on both the background and on large moving objects. In this approach, the motion estimation and stabilization steps are modified compared to the approach outlined above. A flowchart of this new method is depicted in Fig. 2.

In this approach, we obtain a dense motion estimate rather than a global motion estimate. We use optical flow to estimate the correspondence between each pixel in the current input frame and the previous input frame, i.e., the motion vector field. In the next step, we use the estimated motion to calculate the DSR output. This applies to all parts of the image, and thus allows us to apply the same processing to the moving objects and background in the DSR step. Moving objects are no longer detected or treated separately in the DSR step. Therefore, the effects of turbulence are not only mitigated on static scene elements but also on large moving objects whose motion can be estimated using optical flow. However, small moving objects will suffer from motion blur because their motion cannot be accurately estimated.

Several optical flow algorithms were considered for this work:

- The Lucas-Kanade (LK) algorithm [18] for estimating sparse motion vectors on a rectangular grid. The Lucas-Kanade algorithm minimizes the following cost function at multiple image scales using gradient descent, starting at a coarse resolution and successively refining the estimated motion at finer resolution levels:

2 $$ K=\sum \limits_{\mathrm{patch}}\ {\left(\mathrm{\nabla I}\left(\mathrm{i},\mathrm{j},\mathrm{t}\right)\bullet \overrightarrow{u}(t)+I\left(i,j,t\right)-I\left(i,j,t-1\right)\right)}^2, $$

where *I* is the input image, *i* and *j* are spatial indices of pixels, *t* is the frame index, and $\overrightarrow{u}(t)$is the frame-to-frame motion vector. A dense motion field was created by bilinear interpolation of the motion between the sparse motion vectors.

- The Horn-Schunck (HS) algorithm [19], which estimates the entire motion field at once and applies an L2 norm regularization on the gradient of the estimated motion field to suppress outliers. This regularization improves the motion estimation in regions with little image structure compared to the Lucas-Kanade algorithm. The corresponding cost function for this optimization is:

3 $$ K=\sum \limits_{\mathrm{image}}\kern0.5em {\left(\mathrm{\nabla I}\left(\mathrm{i},\mathrm{j},\mathrm{t}\right)\bullet \overrightarrow{u}\left(i,j\right)+I\left(i,j,t\right)-I\left(i,j,t-1\right)\right)}^2+\upalpha \left({\left|\nabla {\mathrm{u}}_{\mathrm{x}}\right|}^2+{\left|\nabla {u}_y\right|}^2\right), $$

where *u*_*x*_ and *u*_*y*_ are the horizontal and vertical components of the motion vector $\overrightarrow{u}\left(i,j\right)$.

- The total variation L1 regularization (TV-L1) algorithm [20], which applies an L1 norm regularization on the gradient of the estimated motion field. Compared to L2 regularization, this allows for a better segmentation between the motion of moving objects and background. The corresponding cost function for this optimization is:

4 $$ K=\sum \limits_{\mathrm{image}}\kern0.5em \lambda \mid \nabla I\left(i,j,t\right)\bullet \overrightarrow{u}\left(i,j\right)+I\left(i,j,t\right)-I\left(i,j,t-1\right)\mid +\mid \nabla {u}_x\mid +\mid \nabla {u}_y\mid . $$

Different optical flow estimation algorithms could be applied as well. However, the higher accuracy that can be achieved with more advanced algorithms typically comes at the expense of a vastly increased computational complexity, which is rather prohibitive for the prospects of a real-time application of this turbulence mitigation method. Also, it should be noted that standard benchmarks for optical flow algorithms typically do not consider turbulence [21, 22], so highly ranking algorithms may not exhibit similar performance under turbulence conditions.

The smoothness of the optical flow field is determined by the parameters controlling the regularization strength of the Horn-Schunck and TV-L1 optical flow algorithms and the patch size of the Lucas-Kanade algorithm. Stronger regularization leads to a smoother field, which reduces inaccuracies due to noise or higher order distortions of local image structures due to turbulence, which vary from one frame to another. However, this also leads to less sharp transitions between the estimated apparent motion of moving objects and the background. Also, the motion of small moving objects may be suppressed entirely in favor of a smooth motion field estimate. The trade-off between suppression of inaccuracies due to noise and distortions and excessive smoothing means that practically, accurate motion estimation required for enhancement of moving objects is only possible if they have linear dimensions on the order of tens of pixels or more under medium turbulence conditions.

The output of the sharpening step consists of sharp images with reduced noise levels whose contents are aligned with the raw frames, both on the moving objects and the background. This implies that the background in these images follows the movement of the turbulence-induced shifts in the raw data. However, for typical detection, recognition or identification tasks this movement is unwanted. It not only causes moving objects to stand out less from the background but also objects’ shapes are dynamically deformed and thus more difficult to interpret. Therefore, good performance on these tasks requires that these shifts are suppressed such that rigid objects and background do not show dynamic deformations. This necessitates a modification of the previous global image stabilization method. Therefore, we suppress these shifts by applying a local stabilization instead of a global stabilization. We apply a temporal autoregressive filter to the frame-to-frame motion to obtain a dynamic reference and warp the sharpened DSR output to this reference. If $\overrightarrow{x}\left(i,j,t\right)$ describes a point in the DSR output for frame *t* with spatial indices *i* and *j*, and the motion estimate indicates that in the previous frame this point was located at position $\overrightarrow{x}\left(i,j,t\right)-\overrightarrow{u}\left(i,j\right)$, then this point is warped to the stabilized position ${\overrightarrow{x}}_s$:

5 $$ \overrightarrow{x_s}\left(i,j,t\right)=\left(1-g\right)\cdotp {\overrightarrow{x}}_s\left(i-{u}_x\left(i,j\right),j-{u}_y\left(i,j\right),t-1\right)+g\cdotp \overrightarrow{x}\left(i,j,t\right), $$

where *u*_*x*_ and *u*_*y*_ are the horizontal and vertical components of the motion vector $\overrightarrow{u}\left(i,j\right)$and *g* is an update rate parameter which determines the amount of motion smoothing and thus the suppression of turbulence induced shifts. However, for fast moving objects, this simple filter may result in a substantial lag between the current location of objects in the input frames and their location in the stabilized output frames. Therefore, the update rate *g* of the filter is increased in each pixel based on the magnitude of the lag ${\overrightarrow{x}}_s\left(i,j,t\right)-\overrightarrow{x}\left(i,j,t\right)$ in that pixel in the previous frame:

6 $$ g={g}_{bg}+\left(1-{g}_{bg}\right)\cdotp {g}_{fast}\cdotp \left(1+\exp \left(4\cdotp \Big(1-2{\left\Vert {\overrightarrow{x}}_s\left(i,j,t-1\right)-\overrightarrow{x}\left(i,j,t-1\right)\right\Vert}_2/\updelta \right)\right)\Big){}^{-1}, $$

Here, *g*_*bg*_ and *g*_*fast*_ are two parameters which determine the update rate for respectively background pixels and pixels which exhibit a lag compared to the raw frame, possibly because they correspond to moving objects in the scene. The scheme in Eq. 6 can be interpreted as a logistic classifier of pixels as belonging to moving object or background based on the lag in the previous frame, and uses this to assign an update rate. The user-adjustable parameter δ specifies the maximum lag above which pixels almost certainly contain moving objects. The choice of the parameter δ balances the stability of the background with lags of moving objects. For higher degrees of turbulence, the apparent motion in the background will appear larger and thus δ should be larger to limit the remaining background motion after stabilization. The stabilization scheme with a fixed update rate *g* will be referred to as local unadaptive stabilization and the stabilization with an update rate set according to Eq. 6 as local adaptive stabilization.

A final challenge for turbulence mitigation relates to occlusions in image sequences. In complex scenes, moving objects such as vehicles or persons may be occluded by more proximate scene elements, such as buildings or foliage for example. For such complex scenes, the divergence of the optical flow between the previous and current frame is an indicator of the areas in the image where occlusions occur, since such a divergence would point to compression or expansion of objects if it would describe the physical motion of those objects whereas in practice objects are typically rigid. However, we find in practice that using the local variance of the optical flow field as a proxy for the divergence provides a more robust indicator of occlusion areas.

In these areas, artifacts in the DSR output due to occlusions are compensated for by replacing the DSR output by a weighted average of the DSR output and the raw input frame according to the following weighting scheme:

7 $$ I\left(i,j,t\right)=\left(1-w\left(i,j,t\right)\right)\cdotp {I}_{raw}\left(i,j,t\right)+w\left(i,j,t\right)\cdotp {I}_{dsr}\left(i,j,t\right), $$

8 $$ w\left(i,j,t\right)=\exp \left(-{V}_x\left(i,j,t\right)/{V}_{max}\right). $$

Here, *V*_*x*_(*i*, *j*, *t*) is the variance in the estimated horizontal motion *u*_*x*_(*i*, *j*) in a rectangular window of 12 × 3 pixels centered around $\overrightarrow{x}\left(i,j\right)$. The vertical component of the estimated motion is disregarded, as large vertical motion in the image corresponds to rapid changes in distance or height of physical objects relative to their surroundings which is uncommon at the large viewing distances to which turbulence mitigation is typically applied.

## Results and discussion

### Turbulence mitigation results

To evaluate the proposed approach for turbulence mitigation, we will consider a number of visible light recordings of real-world scenes. For these scenes, we will investigate whether our proposed dynamic turbulence mitigation can improve the in image quality on moving objects compared to the previous global turbulence mitigation approach, while retaining a similar image quality on the background. Image quality in this case depends both on the visual sharpness of the images and on the level of artifacts, for example due to motion blur or ghosting. However, turbulence-free reference imagery is not available for these real-world scenes, which prohibits a reference-based measurement of image quality on the moving objects. To our knowledge, there are currently also no simulations that accurately reproduce the full complexity of the local image distortions due to turbulence and their spatiotemporal correlation patterns for a high-quality reference video with moving objects either. This prohibits a representative evaluation based on synthetic data. On the other hand, typical no-reference image quality metrics rely on measurement of only edge sharpness. Since a sharpening filter such as the one we apply in our processing can arbitrarily sharpen edges at the expense of noise amplification and ringing artifacts, such a measurement is of limited utility in quantifying improvements. Therefore, the evaluation of our methods will be limited to a qualitative evaluation of the image quality. A further evaluation of all the proposed methods can be found in Section 5, where they are compared on their ability to address the main criteria determining the image quality of the reconstructed images on all datasets in this paper.

#### Sea scenario

**Fig. 3:**
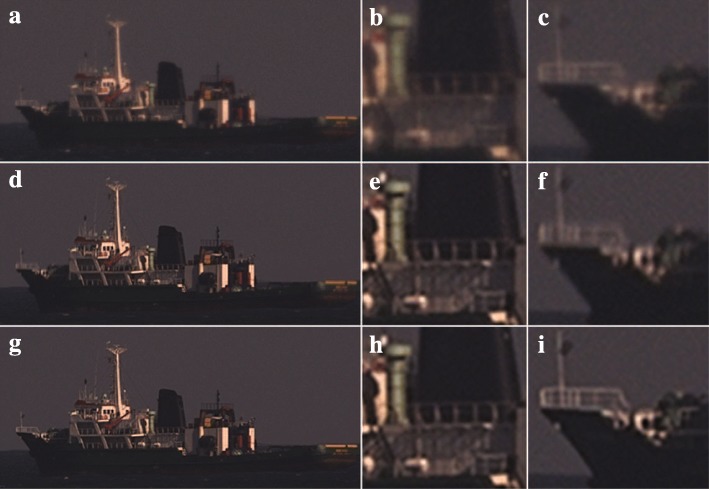
Turbulence mitigation on ship at sea. The ship is about 550 × 210 pixels in size. The top row shows a still from the raw video (**a**) with enlarged cut-outs on the center (**b**) and bow (**c**) of the ship. The middle row (**d**-**f**) shows the results for our turbulence mitigation method from Dijk et al. [16] and the bottom row (**g**-**i**) for the proposed dynamic turbulence mitigation with Lucas-Kanade flow respectively

In this section, we apply our turbulence mitigation method in a sea scenario. Figure 3a–c shows part of a video frame of a ship which is heaving in the waves imaged under medium turbulence conditions. The video from which this region of interest (ROI) of 600 × 280 pixels taken was acquired from the coast of Scheveningen, The Netherlands, using a 300 mm lens set to f/11 mounted on an Allied Vision Mako G-125C 1/3′′ Color CCD camera operating at a frame rate of 30 frames per second. The ship in this video was located approximately 12 km from the viewing position. Because the motion of the sea background is not the same as that of the ship, the estimation of the ship’s motion required for turbulence mitigation needs to be accomplished on the ship itself. The sea itself does contain image structure in the form of waves which exhibit a different motion from the ship.

Figure 3d–f shows the turbulence mitigation result which is achieved when we apply our turbulence mitigation method from Dijk et al. [16] and estimate a translation-only model for the ship’s motion based on an image patch in the center part of the ship. Although the center of the ship does look sharper after turbulence mitigation, which is particularly apparent in the cut-outs in Fig. 3b, e, the bow and stern of the ship exhibit vertical motion blur. This becomes especially apparent when comparing Fig. 3c and f. When we employ the Lucas-Kanade optical flow estimation across the ship to estimate the apparent *local* motion across the field-of-view, we obtain the result shown in Fig. 3g–i. Particularly, the cut-out in panel i reveals that the optical flow solution removes the vertical motion blur caused by the heaving of the ship.

#### Land scenarios

**Fig. 4:**
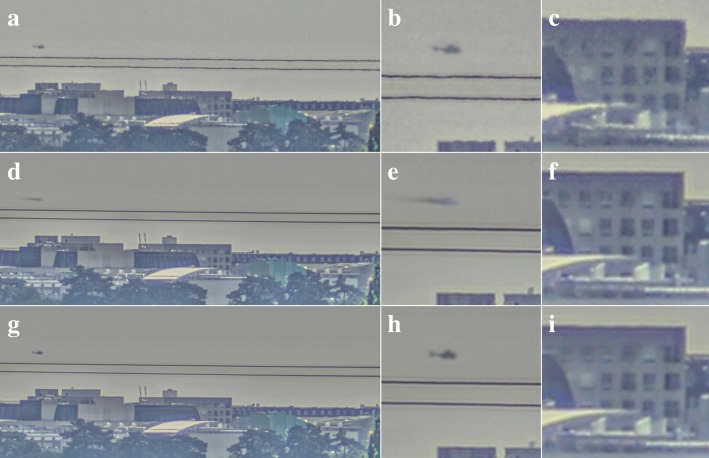
Turbulence mitigation on a helicopter flying over a city. The helicopter is about 55 × 25 pixels in size. The top row shows a still from the raw video (**a**) and cut-outs around the helicopter (**b**) and a building (**c**). The middle row (**d**-**f**) shows the processing results with the global turbulence mitigation method and the bottom row (**g**-**i**) shows the result for dynamic turbulence mitigation with Lucas-Kanade optical flow

We now turn our attention to land scenarios. Unlike in the sea scenario before, land scenarios do provide static backgrounds whose motion can be estimated in image sequences [4]. Figure 4 shows an example of such a sequence containing a helicopter flying above a city imaged under medium turbulence conditions, using a 1000 mm lens set to f/8 mounted on an Adimec TMX6-DHD camera operating at a frame rate of 30 frames per second. Note that the figure only shows an ROI of 912 × 376 pixels from the full frame. The buildings in this video were located approximately 16 km from the camera. The top row of the figure shows a still from the raw imagery after contrast enhancement as well as a cut-out around the helicopter. The detection of the helicopter is complicated by the turbulence-induced deformation of its shape as well as the background noise in the sky. When we apply our global turbulence mitigation, we see in the results in the middle row that the background is indeed greatly enhanced: the wavy roofs of the building now appear sharper and straighter. However, the helicopter was not detected as a moving object because its motion is very similar to the apparent motion in the background due to turbulence. Consequently, the turbulence mitigation algorithm leads to a significant motion blur on the helicopter. This hinders a human observer to detect and recognize the helicopter in the image, even though the noise in the background is greatly reduced. The bottom row of the figure shows the resulting turbulence mitigation when Lucas-Kanade optical flow is used. Evidently, this provides a good mitigation result on the background as well as the helicopter, which appears sharper than in the raw input frame when comparing Fig. 4b and h.

**Fig. 5:**
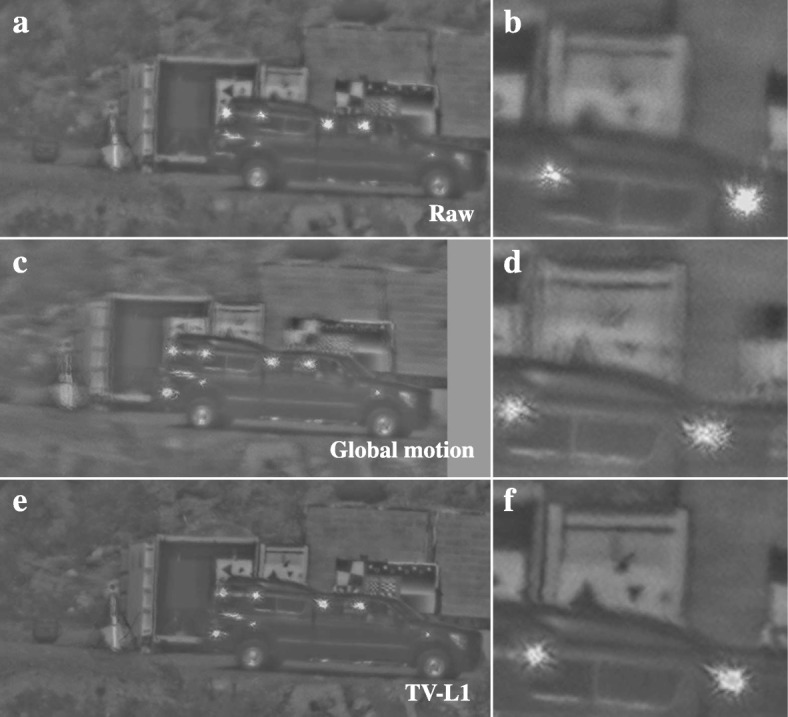
Turbulence mitigation on a moving truck in front of a set of test charts. The top row shows a still from the raw video (**a**) and a cut-out around the top of the truck (**b**). The middle row shows (**c**-**d**) the processing results with the global turbulence mitigation method and the bottom row (**e**-**f**) shows the result for dynamic turbulence mitigation with TV-L1 optical flow. The truck is about 530 × 210 pixels in size

The Lucas-Kanade solution for the motion estimation in the previous case worked fairly well because of the separation between the helicopter and the land. Therefore, the estimated motion in a patch around the helicopter in the image is dominated by the movement of the helicopter. However, in general, this is often not the case for moving vehicles in land scenarios. Figure 5a shows part of a still from an image sequence acquired at the NATO SET226 field trial in Quebec, Canada, using an 800 mm lens set to f/11 mounted on an Allied Vision Mako G-125C 1/3′′ Color CCD camera operating at a frame rate of 30 frames per second and an exposure time of 1.25 ms. Note that this example only uses an ROI of 1016 × 512 pixels from the full frame. It shows a moving truck imaged through medium turbulence (${C}_n^2$ was measured to be around 10^− 14^ m^−2/3^) at a distance of 2.5 km. When we apply our global turbulence mitigation method with a global translation-only motion model, the results shown in Fig. 5c and particularly Fig. 5d reveal a substantial motion blur on the background. Here, the global motion estimator has converged on an estimate that corresponds to the movement of the truck. Local motion estimation using the TV-L1 optical flow provides a much better solution as shown in Fig. 5e, f. This motion estimate leads to good turbulence mitigation both on the vehicle and the background, as witnessed by the sharpness and lack of motion blur in Fig. 5f compared to Fig. 5b, d. Notice in particular the increased sharpness around the windows of the truck, which indicates that the turbulence was mitigated on the moving object itself. Some boundary effects are visible in Fig. 5f at the edge between the top of the truck and the background, where part of the background is erroneously warped in the direction of the truck’s movement.

**Fig. 6:**
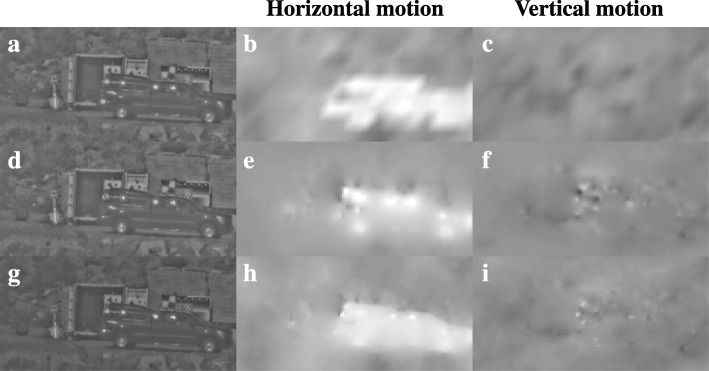
Comparison of three optical flow methods: Lucas-Kanade (**a**–**c**), Horn-Schunck (**d**–**f**), and TV-L1 (**g**–**i**). The left column shows the turbulence mitigation results for the same frame as Fig. 5, whereas the middle and right column show the estimated motion per pixel for that frame expressed in units of pixels per frame

The result in Fig. 5e required an accurate motion estimate on both the truck and the background. We compared three common optical flow estimation algorithms on their ability to achieve this: the Lucas-Kanade algorithm [18], the Horn-Schunck algorithm [19], and the TV-L1 algorithm [20]. Figure 6 shows the turbulence mitigation result obtained with these algorithms in the left column. The middle and right column of this figure shows the estimated motion in respectively the horizontal and vertical direction in the image. These results show that the TV-L1 algorithm provides the sharpest segmentation between the motion of the vehicle and the background, as its horizontal motion field has the sharpest edge at the top of the vehicle. Also, it provides for a fairly homogeneous estimated motion across the truck, as would be expected for a rigid object such as a truck. The Horn-Schunck algorithm has larger inhomogeneities in the estimated motion on the truck due to sun glints on the windows, compared to the Lucas-Kanade and TV-L1 algorithms. This derives from its L2-regularization on the gradients of the estimated motion, which heavily penalizes sharp transitions in the estimated motion per pixel. Therefore, transitions in the estimated motion tend to be smooth around occlusion boundaries or sun glints, even when the local image content in the current frame was not visible in the previous frame and thus no correct motion estimate exists. Thus, we conclude that the TV-L1 algorithm provides a more accurate solution in scenarios such as these where the background exhibits much image contrast right behind the moving vehicle. It should be noted that in some cases, the optical flow solutions do exhibit local outliers in the estimated motion, particularly around repeated patterns in the images, such as the checkerboard charts or the power lines in Fig. 11j. The TV-L1 algorithm appears to be somewhat more vulnerable to this than the other two algorithms.

**Fig. 7:**
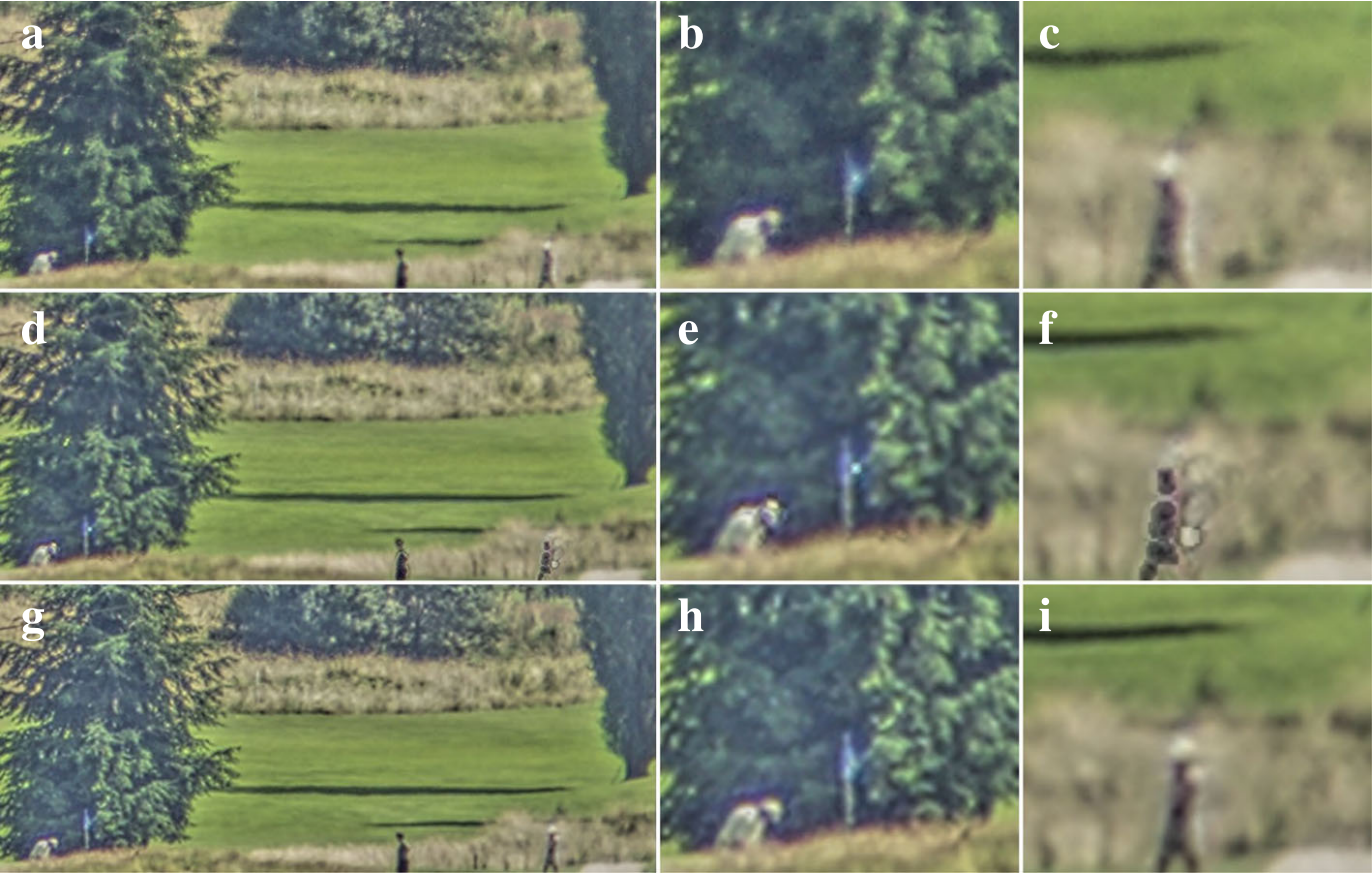
Turbulence mitigation on a golf course scene. The top row shows a still from the raw video (**a**) and two cut-outs around the hole (**b**) and a walking golf player (**c**). The middle row shows the processing results with the global turbulence mitigation method and the bottom row shows the result for dynamic turbulence mitigation with TV-L1 optical flow. The walking golf player is about 25 × 70 pixels in size

Another application of the TV-L1 optical flow algorithm is shown in Fig. 7. Here, the top row shows the raw image which was recorded under medium turbulence conditions, using the same camera and lens as used for Fig. 4. For this application, an ROI of 880 × 408 pixels was used from the full frame. The persons on the golf course in this ROI were located approximately 2.5 km from the camera. The middle row shows the result of our global turbulence mitigation processing with a global translation-only motion model. In this case, we applied less conservative thresholds for moving object detection than for Figs. 3, 4, and 5. Specifically, the maximum difference in the pixel values between raw input frame and the output of the previous DSR step of background pixels was lower here than before. In pixels which are classified as belonging to a moving object, we provide the raw pixel values instead of the DSR result in the output, which forms the input for the sharpening step. Evidently, the movements of the persons in this scene are only partially detected, which is typical under such conditions. The images in the bottom row of the figure show the results of turbulence mitigation using TV-L1 optical flow. A slight motion blur can be observed on the walking person, which may relate to the deviating motion of their swinging arms. However, the moving persons appear sharper in these images, while the turbulence mitigation in the background still performs well, as evidenced by the sharp and straight shadow of the tree to the left.

**Fig. 8:**
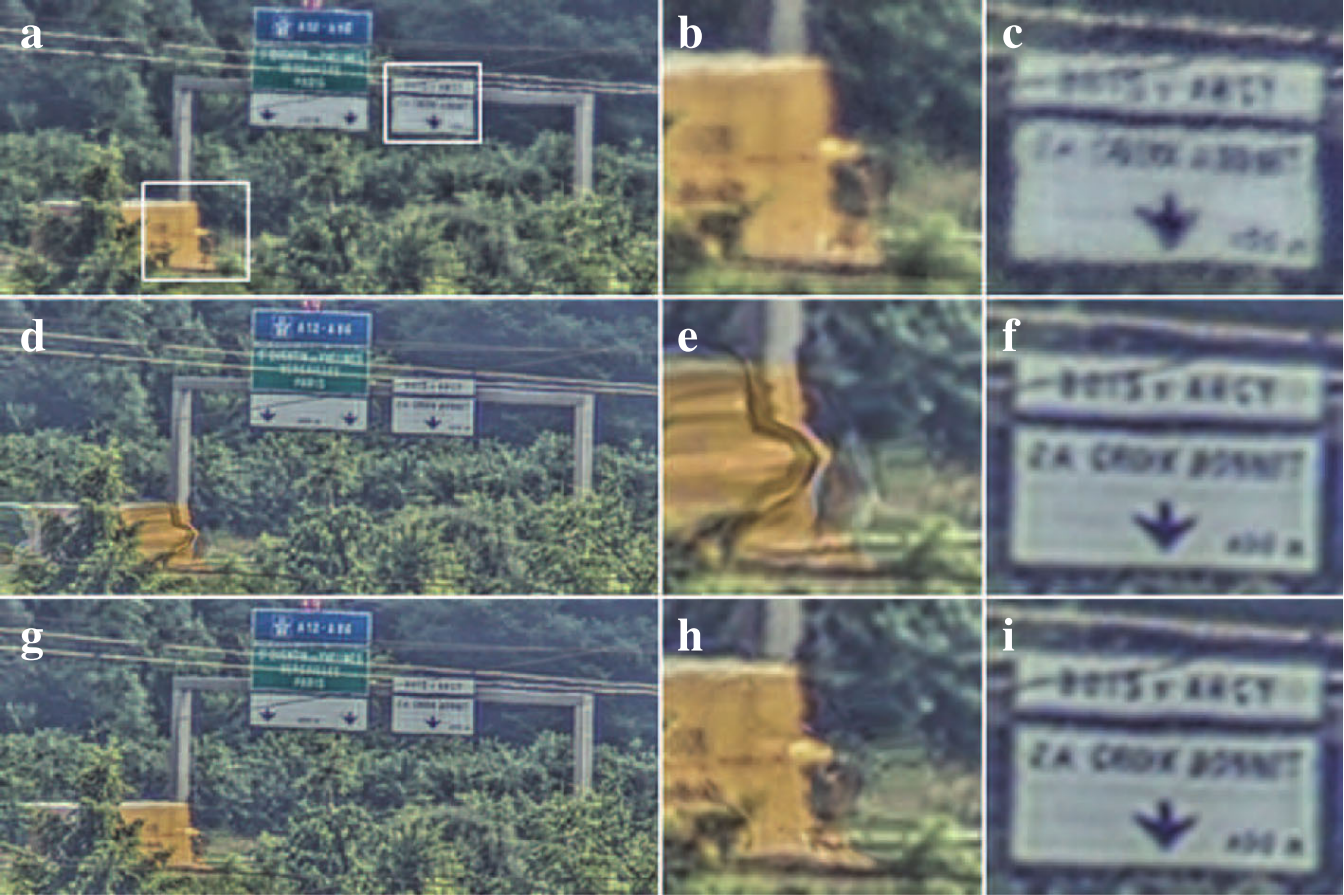
Turbulence mitigation on a highway scene. The top row shows a still from the raw video (**a**) and two cut-outs (**b**-**c**). The middle row shows (**d**-**f**) the processing results for dynamic turbulence mitigation with TV-L1 optical flow without occlusion compensation. The bottom row (**g**-**i**) shows the result when the occlusion compensation is applied. The height of the truck is about 100 pixels

An important challenge for the methods demonstrated thus far is their application to scenes with pronounced occlusion effects. The results so far were obtained without applying an occlusion compensation method to mitigate these effects. Consider for example the turbulence mitigation results in Fig. 8, obtained for a different ROI (of 880 × 416 pixels) from the same video as the one used for the results in Fig. 4. The scene shows a highway with moving vehicles at approximately 3 km from the camera. Figure 8d shows artifacts across the moving truck because of mixing of image contents of the truck and the occluded background. This result from the underlying assumption of the optical flow algorithm used that scene contents in the current frame were visible in previous frames as well. This assumption is violated at the front and back of the truck and around the edges of the tree in front of the truck to the left. In the bottom row of Fig. 8, we reduced these artifacts by modifying the DSR step: in pixels where the local variance of the motion field is high, the DSR output is replaced by a weighted average of the DSR output and the raw input frame. This occlusion compensation does indeed reduce the line artifacts across the truck, as seen in Fig. 8h. However, a distortion in the background due to erroneous image motion estimation can be still seen in front of the truck. What is noteworthy however is the accuracy of the vertical segmentation between moving truck and the background: the structures above are hardly affected by the moving truck.

### Image stabilization results

The turbulence mitigation results in the previous section showed that in many cases the local motion estimation with optical flow provides for sharp output images with reduced noise levels. In this section, we evaluate the performance of the stabilization step in the proposed turbulence mitigation method.

**Fig. 9:**
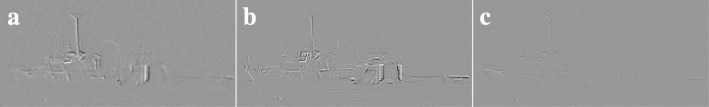
Stabilization results for the ship in Fig. 3. Frame-to-frame differences are shown between the raw frames (**a**), the processing results with a global motion model estimated in the center of the ship (**b**), and for the proposed dynamic turbulence mitigation with Lucas-Kanade optical flow (**c**)

Figure 9 illustrates this effect for the ship shown in Fig. 3. Figure 9a shows the difference between the raw frame in Fig. 3a and the subsequent raw frame, whereas Fig. 9b shows the difference between the output of the sharpening step for that frame and the next frame. Clearly, large frame-to-frame differences are present, indicating substantial apparent movements of all parts of the ship relative to each other even though the ship is a rigid object. Figure 9c shows how this is resolved with the local unadaptive stabilization scheme as detailed in Eq. 5, for a parameter value *g* = 0.05. The frame-to-frame differences in the center of the ship are much smaller. The remaining differences on the bow, stern, and mast of the ship are consistent with the rotational motion of the ship in the waves, and consequently the ship appears rigid in the stabilized output imagery.

**Fig. 10:**
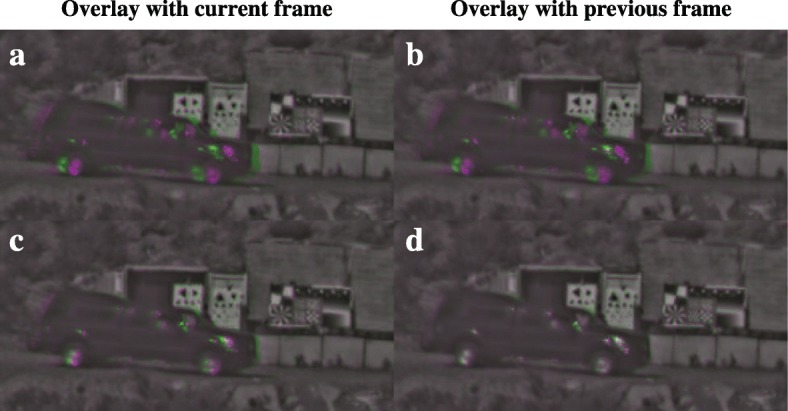
Stabilization result for the truck sequence in Fig. 5. Overlays are shown between the stabilized processing result in magenta and the corresponding raw frame (left) and preceding raw frame (right). In (**a**, **b**), the stabilization does not distinguish between background and moving object. In (**c**, **d**), the stabilization results are shown when the stabilization is modified to take into account moving objects, which leaves an overlay with less color and hence less mismatch between the stabilization output and particularly the previous raw frame

The result in Fig. 9 illustrates the benefit of locally stabilizing the output imagery. However, the ship’s movement is relatively slow. When we consider the moving truck in Fig. 5, it becomes apparent that the stabilization scheme in Eq. 5 may introduce a substantial lag between the current position of the truck and its position in the stabilized output. This is shown in Fig. 10, where Fig. 10a shows the current raw frame in magenta and the output stabilized with the scheme in Eq. 5 with fixed *g* = 0.2 in green. When the raw frame and stabilized frame are locally exactly aligned, the corresponding pixels appear black, white, or gray. Clearly, the truck’s position is lagging substantially behind the current position. In this case, the lag is roughly five frames (data not shown). Figure 10c shows the result when the stabilization scheme is modified to account for moving objects as discussed at the end of Section 2, which is referred to as the local adaptive stabilization scheme. The lag parameter δ was set here to a value of 10. The difference between the raw frame and the stabilization output is much reduced, as evidenced by the apparent reduction in green and magenta regions in the image. Figure 10d shows the previous frame in green instead, and the apparent alignment with the stabilization output of the current frame in magenta shows that the truck’s positions lags by only about 1 frame relative to its current position.

### Comparison of methods across datasets

In the previous sections, we presented different possibilities for mitigating the effects of atmospheric turbulence when imaging several different scenes: several choices in optical flow algorithms, three types of image stabilization, and the possibility to use an occlusion compensation scheme or not. The utility of each method was demonstrated on a different dataset each time. In this section, we will evaluate all methods on each of the datasets to assess their relative performance in each case. In addition, we will provide an overview of how the different methods affect the different determinants of the image quality for the different datasets.

**Fig. 11:**
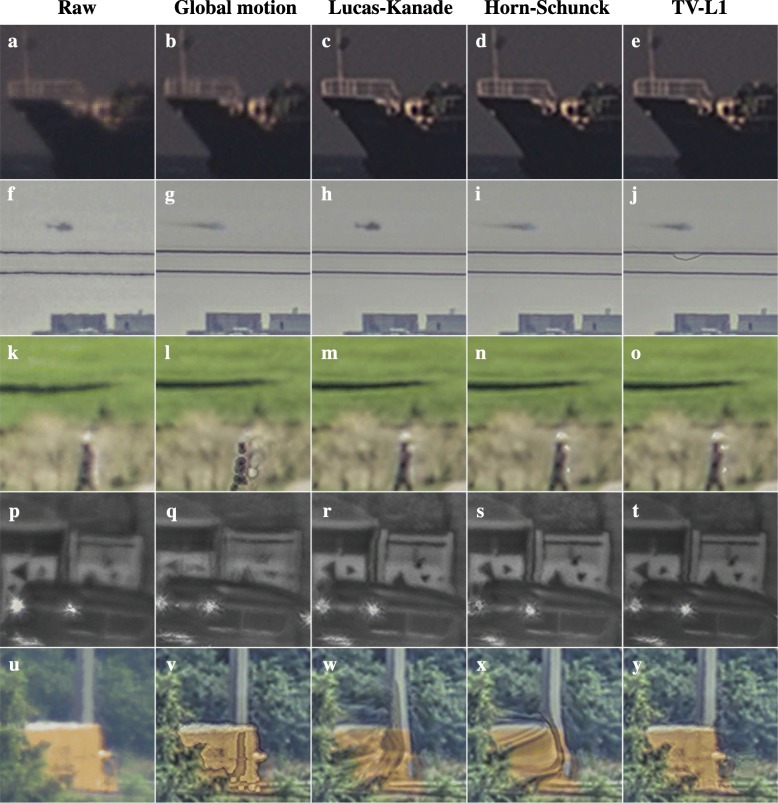
Application of the different motion estimation methods to each of the datasets in this article. Each column (**a**-**y**) shows the results in a representative region of interest for one of the motion estimation methods. The leftmost column shows the corresponding area in the raw input image

Firstly, we will compare the various motion estimation algorithms presented before: global motion estimation with a single motion vector describing only the translation over time in the image sequence, and Lucas-Kanade, Horn-Schunck, and TV-L1 optical flow estimation. The resulting image quality for each of the datasets is demonstrated in Fig. 11. From these results, we can draw several conclusions.

The results for the sea scenario shown in Fig. 11a–e demonstrate that all optical flow methods in Fig. 11c–e obtain a similar image quality. All of them can account for the rotational motion of the ship. Similarly, the optical flow methods show similar performance on the golf course dataset shown in Fig. 11m–o. The key similarity here is the relative lack of occlusions of moving objects or background, because the backgrounds in these cases have limited image structure which may affect the motion estimation on the moving objects. Interestingly, the Lucas-Kanade exhibits the best image quality on the moving helicopter.

Both the results for the Horn-Schunck and TV-L1 algorithms exhibit motion blur on the helicopter. This suggests that the regularization of these algorithms favors a smooth motion field over an accurate motion estimate on the small moving helicopter. Instead, the Lucas-Kanade algorithm, which only uses a small local image patch for motion estimation, is not affected by more distant static scene elements and does not cause motion blur on the helicopter. Figure 11j also illustrates the vulnerability of the TV-L1 algorithm to outliers in the estimated motion, which are sometimes observed near repeated scene elements such as the two power lines. On the other hand, Fig. 11p–y clearly shows that in scenes with complex backgrounds right behind the moving trucks, the TV-L1 algorithm provides a superior image quality. In particular, the TV-L1 shows the least shearing of background structures above the moving vehicles. Additionally, Fig. 11w, x exhibits artifacts due to blending of the moving truck and the signpost in the background, which indicates that the transitions in their motion field were not sharp enough to allow occlusion compensation.

**Fig. 12:**
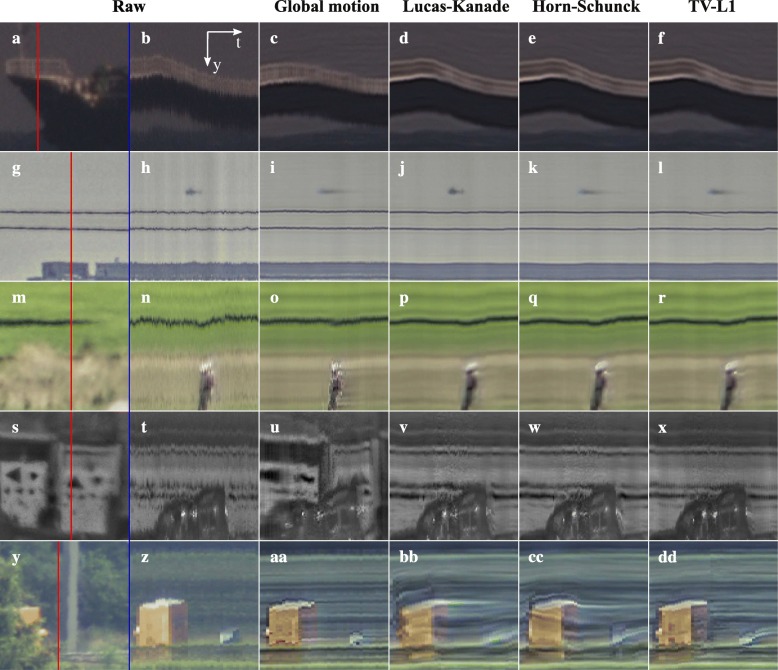
Temporal cross-sections of the different motion estimation methods to each of the datasets in this article (**b**-**f**, **h**-**l**, **n**-**r**, **t**-**x**, **z**-dd). The leftmost column (**a**, **g** **m**, **s**, **y**) shows region of interest in the first frame of each temporal cross section. The red lines indicate the vertical image lines which for which temporal cross-sections are shown in the other columns

To further visualize the impact of the different optical flow methods, Fig. 12 shows temporal cross-sections for a single image line for each of the regions of interest in Fig. 11. These results support the same conclusions as Fig. 11, but further emphasize that the global motion estimation is prone to oversegmentation of moving objects. This refers to the extent to which the moving object detection in our global motion estimation-based method tends to break up the moving objects in the scene into multiple patches, some of which are classified as moving object and some of which are not. The bottom row in Fig. 12 also shows that the TV-L1 algorithm has a clear benefit when it comes to the back of the moving truck, which exhibits less ghosting, i.e., blending of the moving truck into the background image in parts of the image where it has already passed. Finally, these results show that in general the different optical flow methods substantially reduce the turbulence-induced background motion, which can be seen from the lack of wavelike perturbations on the more or less horizontal lines corresponding to static scene elements.

**Fig. 13:**
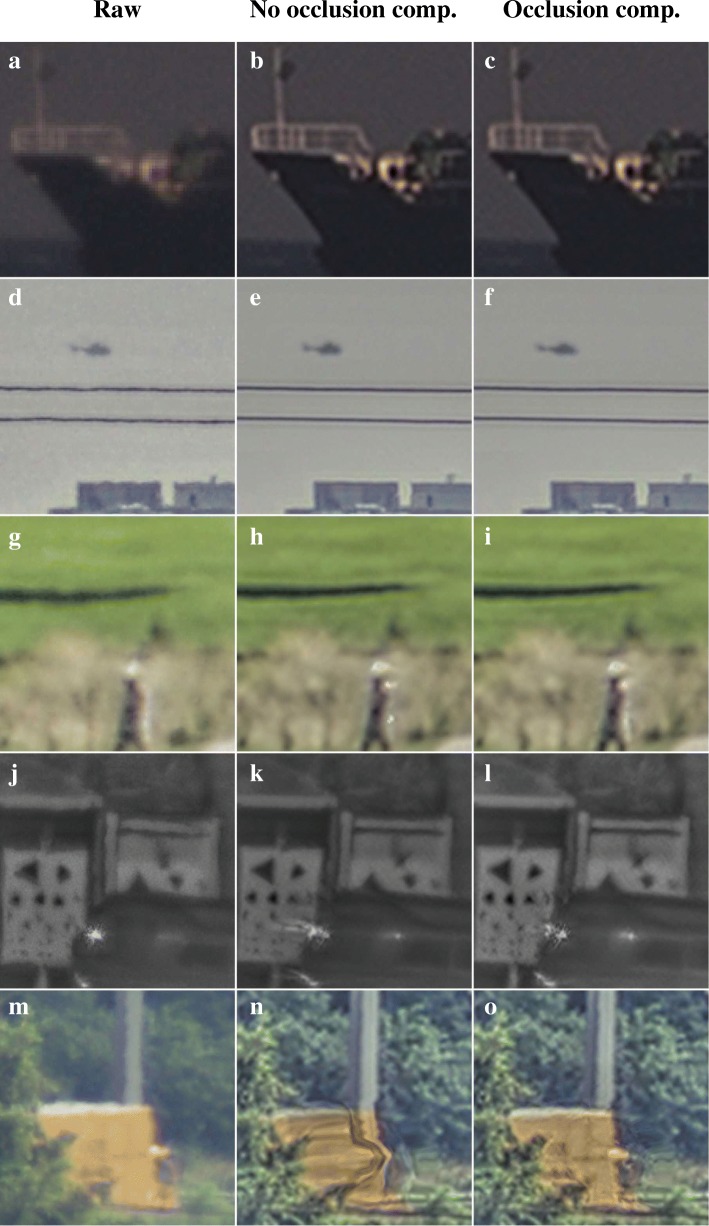
Comparison of turbulence mitigation results with occlusion compensation (right column) and without (middle column). Lucas-Kanade optical flow estimation was used for the datasets shown in subfigures (**a**–**f**) and TV-L1 optical flow estimation for subfigures (**g**–**o**)

The use of occlusion compensation for the different datasets is evaluated in Fig. 13. In this figure, a comparison is made between results obtained with and without occlusion compensation, when the same optical flow algorithms are used for each dataset as in the preceding sections. The results in Fig. 13a–i suggest that in the absence of occlusions in the imagery, the application of occlusion compensation has little effect on the resulting image quality. No artifacts show up in images where the compensation is applied in the absence of an occlusion. However, occlusion compensation does prevent artifacts due to streaking of the sun glint on the moving truck in Fig. 13l and blending of the moving truck and the background in Fig. 13o. Therefore, we conclude that the proposed occlusion compensation seems to provide a robust means of improving image quality.

**Fig. 14:**
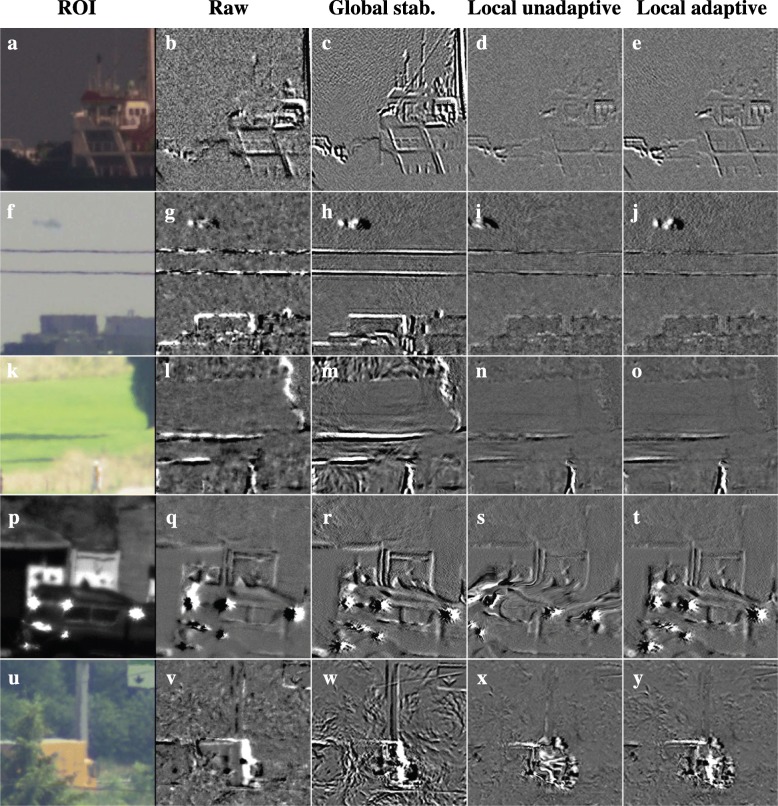
Comparison of stabilization results. The left column (**a**, **f**, **k**, **p**, **u**) shows the region in the raw frame for which results are visualized per row. Subsequent columns show the difference between the previous frame and current frame for respectively the raw frames (**b**, **g**, **l**, **q**, **v**), global stabilization (**c**, **h**, **m**, **r**, **w**), local unadaptive stabilization with a fixed update rate according to Eq. 5 (**d**, **i**, **n**, **s**, **x**), and local adaptive local adaptive stabilization with a variable update rate according to Eq. 6 (**e**, **j**, **o**, **t**, **y**). Each difference image has the same normalization as the other difference images in the same row of the figure

**Fig. 15:**
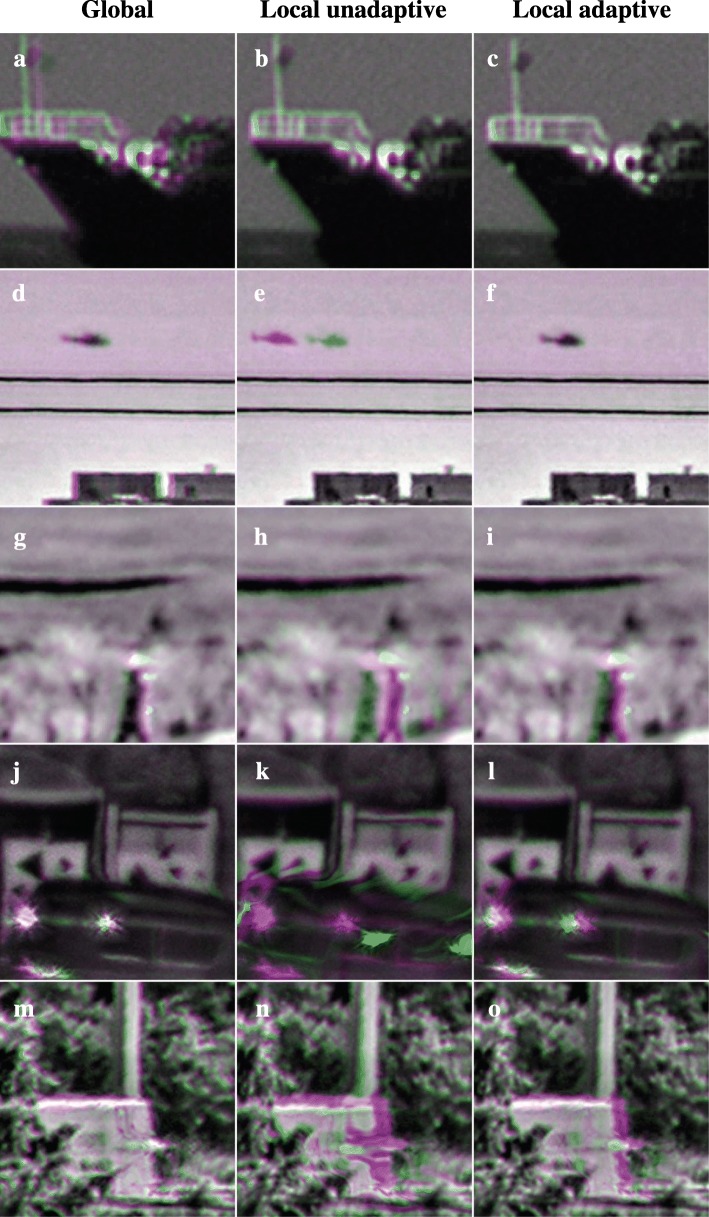
Comparison of stabilization results. The columns show the difference between the current output (magenta) and raw input frame (green) for respectively global stabilization (**a**, **d**, **g**, **j** ,**m**), local unadaptive stabilization with a fixed update rate according to Eq. 5 (**b**, **e**, **h**, **k**, **n**), and local adaptive local adaptive stabilization with a variable update rate according to Eq. 6 (**c**, **f**, **i**, **l**, **o**)

The different methods for motion stabilization are compared in Figs. 14 and 15. Like Figs. 9 and 10, the effect of the stabilization is visualized in terms of frame-to-frame differences between output images and in terms of the difference between output frames and corresponding raw input frames. Frame-to-frame differences are used to assess whether apparent variations in the background over time due to turbulence are suppressed. Accurate stabilization should show few frame-to-frame differences on the static background. Both the local unadaptive and local adaptive stabilization obtain a better result on this criterion than the raw frames or the global stabilization scheme. However, Fig. 14s and Fig. 15k does show that the unadaptive stabilization causes a curved deformation on the upper part of the moving truck. These deformations are caused by inaccuracies in the motion estimation at the top of the truck in some of the previous frames, likely due to the sun glints.

Figure 15 shows that the local adaptive stabilization scheme causes a much smaller lag between the moving objects in the output frames and raw input frames than the local unadaptive stabilization: the right column shows a much smaller mismatch between the positions of the moving vehicles in the magenta and the green color channel than the middle column. Taking all these results into account, we conclude that the local adaptive stabilization scheme is generally to be preferred over the other stabilization methods, as it provides good results over wider range of conditions.

The results discussed so far show that there are a variety of different factors that determine the perceived image quality of the turbulence mitigation outputs. These factors include both the general sharpness and stability of static background elements of scenes and various artifacts caused by processing of moving objects. Based on the results in the preceding paragraphs and sections, we have summarized the factors influencing our assessment of the perceived image quality into a set of more general categories. To obtain an independent evaluation of the relative performance of each method on these categories, we performed an observer experiment with three test subjects from Adimec, a possible customer for the techniques reported here: two optical designers and one image processing expert. The subjects, although active in the field of imaging with atmospheric turbulence, were not otherwise involved in the research reported here and could thus independently assess the different methods. Subjects were first shown all results videos for all methods. Next, they were shown all result videos of one of the methods, without indication of which methods was used, and were asked to independently and without deliberation write down a single score on each category for all videos. In particular, they were asked to score the performance of each method relative to the raw input images before mitigation on a five point scale, where performance was expressed in terms of their ability to perceive the scene in regions of the scenes which could be affected by influences from each category. Table 1 shows the median result of the test subjects’ scores for all categories and methods. Note that in this table, one of the scoring criteria is the oversegmentation of moving objects. This refers to the extent to which the moving object detection in our global motion estimation-based method tends to break up the moving objects in the scene into multiple patches, some of which are classified as moving object and some of which are not.

**Table 1:** Comparison of methods on the main factors determining the image quality. Three independent test subjects scored the performance, in terms of their ability to perceive the scene, relative to the raw input images before mitigation as: large improvement [++], improvement [+], no significant improvement or deterioration [o], deterioration [−], and large deterioration [−−]. High scores on oversegmentation and deformation thus indicate positive contributions to image quality. The results for the different motion estimation methods (LK, HS, TVL1) are reported assuming local adaptive stabilization but not occlusion compensation. The results for dynamic turbulence mitigation with stabilization or occlusion compensation are reported assuming the best performing motion estimation method for a given scene

	Sharpness of static background	Sharpness of large moving objects	Sharpness of small moving objects	Oversegmentation of moving objects	Ghosting on or around moving objects	Image shearing on motion boundaries	Dynamic deformation of scene	Lag of moving objects
Global motion + stab.	+	+	+	–	–	–	+	o
Dynamic turb mit. + LK	+	o	+	o	–	–	+	o
Dynamic turb mit. + HS	++	o	o	o	–	–	+	o
Dynamic turb mit. + TVL1	+	+	+	o	o	––	+	o
Dynamic turb mit. + global stab.	+	+	+	o	o	o	+	o
Dynamic turb mit. + local unadaptive stab.	+	o	–	o	o	––	––	o
Dynamic turb mit. + local adaptive stab.	++	+	o	o	–	o	++	o
Dynamic turb mit. + local adaptive stab. + occlusion compensation	+	+	o	o	o	o	–	o
Fishbain et al.	o	o	o	o	o	o	o	o
Oreifej et al.	–	+	–	––	––	o	+	o

The scores in Table 1 show several noteworthy results. Firstly, our new proposed methods do indeed seem to resolve the negatively scored aspects of our previous global motion estimation-based method. However, against our expectations, the TVL1 motion estimation receives a strongly negative on image shearing, even though most of the results for stabilization and occlusion compensation were also obtained with that motion estimation. The test subjects do positively evaluate the local adaptive stabilization compared to the other types of stabilization. The unadaptive stabilization results in spatially varying lag across moving objects. This variable lag was interpreted as a dynamic deformation which was both more unfavorable than the lagging average position of moving objects and more unfavorable than the deformation from the turbulence induced shifts. Based on the score for global stabilization, it appears that the remaining deformation after local averaging of small-scale turbulence effects was not deemed to be very consequential by the subjects. It remains unclear why the occlusion compensation was ranked negatively on the presence of dynamic deformation. The most notable difference compared to the results obtained with dynamic turbulence compensation and local adaptive stabilization only is the reduction in ghosting artifacts around occlusion boundaries. This reduction is also attested to by the improved score on the ghosting on or around moving objects. A possible explanation may be that the order of displayed results affected on which parts of which videos the subjects focused their attention. In summary, the general picture that emerges from these results is that the changes from global turbulence mitigation to dynamic turbulence mitigation and from global to local adaptive stabilization are positively evaluated by the subject, but that the choice of preferred local motion estimation method and the utility of using occlusion compensation are not ambiguous and may depend on the specific application.

### Comparison with existing methods in the literature

The results so far demonstrated the performance of the methods proposed in this paper on various scenarios. In this section, we make a comparison between these methods and two methods representing the current state-of-the-art in turbulence mitigation in the literature: the approach by Fishbain et al. [8], which is considered as a representative for methods that aim to detect moving objects based on displacements relative to a reference frame, and the recent three-way decomposition approach by Oreifej et al. [12] For more details on these methods, the reader is referred back to Section 1. For the Fishbain approach, the data of the published results are available, but the source code for their method is not. For the Oreifej approach, both data and source code are available, but since their data only contain very small targets, they are not relevant for evaluation of turbulence mitigation with large moving objects. In our application of the Oreifej approach, the source code was applied with default parameters. No retraining was undertaken of the parameters of the classifier which is used in this approach to initialize the confidence of pixels belonging to background or moving objects. For brevity, we only show results of our dynamic turbulence mitigation methods with the previously best performing motion estimation, adaptive stabilization and occlusion compensation.

**Fig. 16:**
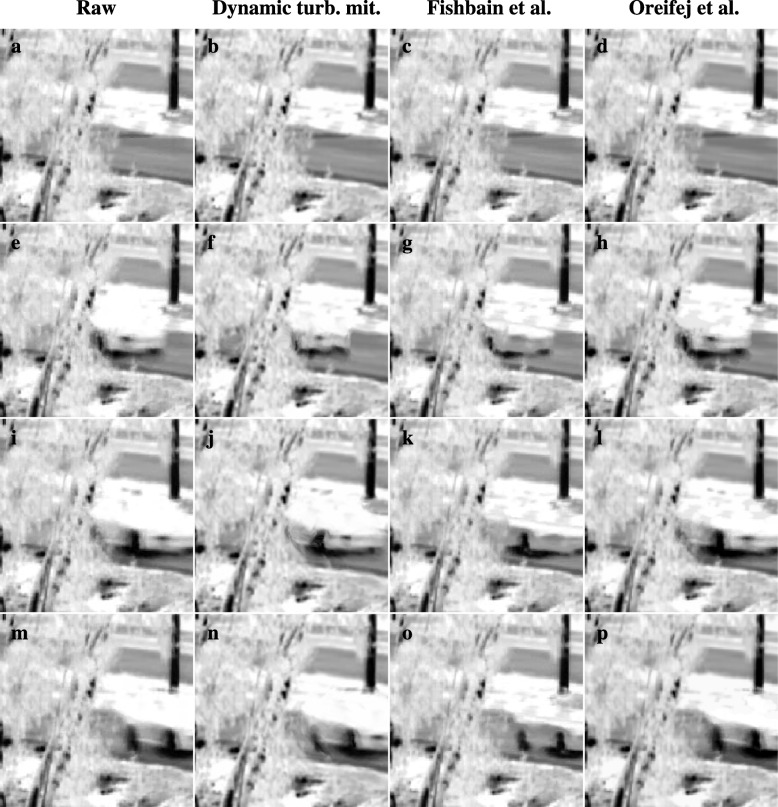
Comparison of turbulence mitigation results on a thermal infrared recording of a car appearing from behind a set of trees. The different rows show different frames in the sequence. Columns show respectively from left to right: raw frames (**a**, **e**, **i**, **m**), results obtained with our method (**b**, **f**, **j**, **n**), results obtained by Fishbain et al. [9] (**c**, **g**, **k**, **o**), and results obtained with the method by Oreifej et al. [13] (**d**, **h**, **l**, **p**)

Figure 16 shows the turbulence mitigation results on several frames of a challenging video from the data Fishbain et al. The video shows a thermal infrared recording of a car appearing from behind a set of trees. The intensity of the car in the imagery is similar to the ground in between the two lanes of the road. This lack of contrast causes the background to bleed through in the top part of the car in the results of Fishbain et al. (third column from the left) and the three-way decomposition approach. (right column), but much less so in the results of our dynamic turbulence mitigation (second column from the left). However, the three-way decomposition approach performs well on the bottom part of the car, which has a high contrast with the road, whereas our dynamic turbulence mitigation in Fig. 16f results exhibit artifacts near the occlusion boundary between the trees and the car. This indicates that the motion estimation and occlusion compensation were inaccurate.

**Fig. 17:**
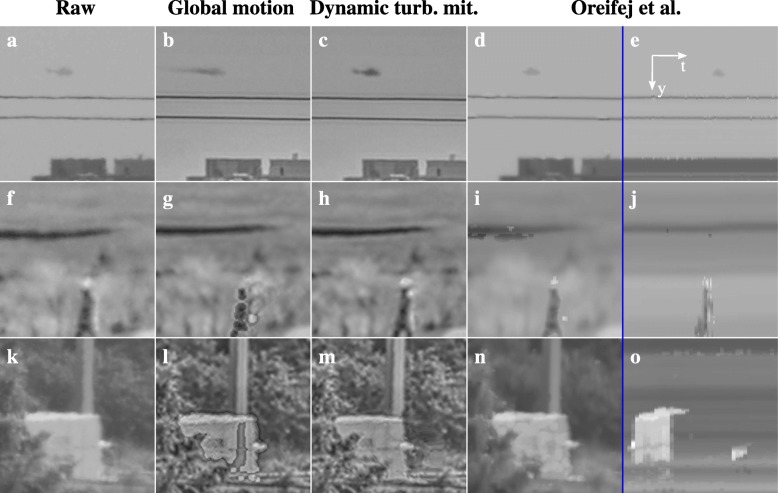
Comparison of turbulence mitigation results on grayscale imagery of a golf course scene, scene with a helicopter flying over a city, and highway scene. Columns show respectively from left to right: raw frames (**a**, **f**, **k**), results obtained with our global turbulence mitigation method (**b**, **g**, **l**), results obtained with our dynamic turbulence mitigation with TV-L1 optical flow and occlusion compensation (**c**, **h**, **m**), and results obtained with the three-way decomposition approach shown as images (**d**, **i**, **n**) and temporal cross-sections (**e**, **j**, **o**)

Because of the available source code, the three-way decomposition approach can also be compared to our dynamic turbulence mitigation method on datasets presented in this manuscript. Such a comparison is made in Fig. 17, which shows respectively from left to right the raw input frames, results of our global turbulence mitigation, results of our dynamic turbulence mitigation method, and results obtained with the three-way decomposition approach. The figure shows results on grayscale converted images rather than on the original color images. The latter lead to artifacts in the three-way decomposition approach, due to different assignments of which pixels contain moving objects in the different color channels. The results show that the three-way decomposition approach detects more of the moving object pixels than the global turbulence mitigation approach, but also incorrectly identifies regions in the images with strong edges as moving objects, such as the power lines in the top row of the figure. Moreover, the background in the results obtained with the three-way decomposition approach mostly appears stable in the temporal cross-sections in the rightmost column of Fig. 17, but much less sharp than in the results obtained with our global and dynamic turbulence mitigation methods.

When combining the above comparisons, we conclude that no single method performs best on all datasets. The three-way decomposition approach performs slightly better on the video from Fishbain et al., whereas our dynamic turbulence mitigation approach exhibits fewer artifacts in Fig. 17. The most evident difference between these datasets is the availability of fine details in the images to enable accurate optical flow estimates. This is essential for the performance of our dynamic turbulence mitigation approach, particularly for accurate occlusion compensation. However, a more elaborate comparison on more datasets would be needed to identify the circumstances in which either method is favored over the other. It should be noted here that the three-way decomposition approach only detects moving objects in the images and is thus not able to mitigate the effects of turbulence on those objects themselves, such as the ship in Fig. 3. Conversely, our dynamic turbulence mitigation method is not able to accurately mitigate the turbulence on the small moving objects in the data shown in Oreifej et al. [12] to evaluate their three-way decomposition method.

The performance of the methods of Fishbain et al. and Oreifej et al. has also been scored in Table 1, similar to our own methods. These scores mostly match our expectations. Most significantly, however, the test subjects did not notice substantial negative impacts from the ghosting and oversegmentation artifacts of the method by Fishbain et al. This is attributed to the relative low contrast between moving cars and background for the single video on which this method was assessed and may not generally hold if the method could be applied to other data.

## Conclusion

In summary, we propose a new approach for dynamic turbulence mitigation on both the background and on large moving objects. Large moving objects here are considered to have linear dimensions on the order of tens of pixels or more, such that their motion can be accurately determined using the discussed optical flow methods. In our approach, we modify our previous global turbulence mitigation method in three ways. Firstly, we replace the global motion estimation with optical flow motion estimation that provides an estimate of the frame-to-frame motion for each pixel, where different choices are possible for the optical flow algorithm. Secondly, we introduced a method to compensate for occlusion effects in the imagery. Thirdly, we introduced local image stabilization methods to suppress deformation of both moving objects and background: a simple temporal autoregressive filter of the frame-to-frame motion and a modification of this filter that accounts for moving objects.

The proposed method enables turbulence mitigation not only on static backgrounds but also on certain moving objects, in contrast to most previous turbulence mitigation methods in the literature which only aim to detect moving objects and represent them by the raw pixel values in output imagery. In a direct comparison, our methods exhibit competitive performance relative to the representative state-of-the-art methods of Fishbain et al. [8] and Oreifej et al. [12] on challenging datasets. Small moving objects, such as the helicopter in Fig. 4, may suffer from motion blur in our method, because the motion cannot be accurately determined using the optical flow algorithms considered in this paper. To the best of our knowledge, the approach by Anantrasirichai et al. [13] is the only other published method designed for turbulence mitigation on moving objects. We expect that the advanced fusion scheme employed in this method may be beneficial for the background sharpness. Conversely, our method likely performs better on large moving objects because of the lack of a hard classification of pixels into moving objects and background, which is key benefit compared to the method of Fishbain et al. [8], and the motion estimation which was optimized for performance on moving objects.

We qualitatively evaluated the new approach on both sea and land scenarios. For the ship at sea, the approach showed good performance in terms of sharpness as well as stability on the heaving ship. Also, for the land scenarios, we qualitatively demonstrated improved turbulence mitigation on moving objects compared to our previous global approach, while retaining a good performance on the background. In particular, we demonstrated that increased sharpness could be achieved on a heaving ship, a flying helicopter, and a moving truck. Our results on the data showing the walking golf player and vehicles on the highway did not show significant improvements on image quality on the moving objects, but also no substantial degradation due to artifacts.

The choice of optical flow algorithm depends on the application. The Lucas-Kanade algorithm shows good turbulence mitigation results for scenes with limited occlusion of moving objects or background, such as the heaving ship at sea or the flying helicopter. The more computationally expensive TV-L1 optical flow typically provides much better segmentation between the motion of large moving objects and background, as shown on the driving car and golf players. Conversely, the Lucas-Kanade algorithm shows more accurate motion estimation on the small moving objects such as the helicopter than the TV-L1 optical flow. It appears that in the helicopter case, the TV-L1 optical flow prefers a smooth solution where the estimated motion is the same for the background and the small moving object, rather than a field where the estimated motion on the helicopter differs from the background. In some cases, the TV-L1 solution also exhibits local outliers in the estimated motion, particularly around repeated structures in the images. Taken together, we conclude that in terms of image quality, the faster Lucas-Kanade algorithm is preferred for small moving objects and simple backgrounds, whereas the TV-L1 algorithm is preferred for large moving objects and complex backgrounds.

Occlusion events provide one of the greatest challenges for the application of dynamic turbulence mitigation. Future work will therefore focus on accurately identifying occlusions and accounting for them in the image restoration and stabilization, for example when vehicles disappear behind buildings. Another challenge which will be addressed in the future work is to investigate how motion blur may be prevented on small moving objects, whose motion could not be accurately estimated using the optical flow considered in this paper.

## Abbreviations

DSR: Dynamic super-resolution
HR: High resolution
HS: Horn-Schunck
LK: Lucas-Kanade
PSF: Point spread function
ROI: Region of interest
TV-L1: Total variation L1 regularization
